# [Retracted] MicroRNA-122 affects cell aggressiveness and apoptosis by targeting PKM2 in human hepatocellular carcinoma

**DOI:** 10.3892/or.2023.8475

**Published:** 2023-01-02

**Authors:** Qiuran Xu, Meiqi Zhang, Jianfeng Tu, Linxiao Pang, Wenwei Cai, Xin Liu

Oncol Rep 34: 2054–2064, 2015; DOI: 10.3892/or.2015.4175

Following the publication of both the above article and a corrigendum (doi: 10.3892/or.2021.8073) that was concerned with the correction of overlapping data panels in Figs. 6 and 7, it has been drawn to the Editors' attention by a concerned reader that the proposed replacement cell invasion assay shown in the revised version of Fig. 7A, and also flow cytometric data featured in Fig. 5A and C, were strikingly similar to data appearing in different form in other articles by different authors. Owing to the fact that these contentious data in the above article had already been published elsewhere, or were already under consideration for publication, prior to its submission to *Oncology Reports*, the Editor has decided that this paper should be retracted from the Journal. The authors were asked for an explanation to account for these concerns, but the Editorial Office did not receive a reply. The Editor apologizes to the readership for any inconvenience caused.

